# Nicotinic acetylcholine receptor variants associated with susceptibility to chronic obstructive pulmonary disease: a meta-analysis

**DOI:** 10.1186/1465-9921-12-158

**Published:** 2011-12-17

**Authors:** Jing Zhang, Hanssa Summah, Ying-gang Zhu, Jie-Ming Qu

**Affiliations:** 1 Department of Pulmonary Medicine, Zhongshan Hospital, Shanghai Medical College, Fudan University, Shanghai 200032, China; 2 Department of Pulmonary Medicine, Huadong Hospital, Shanghai Medical College, Fudan University, Shanghai 200040, China

**Keywords:** Chronic Obstructive Pulmonary Disease (COPD), Nicotine acetylcholine receptor (nAChR), *CHRNA -; *, Single nucleotide polymorphism (SNP)

## Abstract

**Background:**

Only 10-15% of smokers develop chronic obstructive pulmonary disease (COPD) which indicates genetic susceptibility to the disease. Recent studies suggested an association between COPD and polymorphisms in *CHRNA *coding subunits of nicotinic acetylcholine receptor. Herein, we performed a meta-analysis to clarify the impact of *CHRNA *variants on COPD.

**Methods:**

We searched Web of Knowledge and Medline from 1990 through June 2011 for COPD gene studies reporting variants on *CHRNA*. Pooled odds ratios (ORs) were calculated using the major allele or genotype as reference group.

**Results:**

Among seven reported variants in *CHRNA*, rs1051730 was finally analyzed with sufficient studies. Totally 3460 COPD and 11437 controls from 7 individual studies were pooled-analyzed. A-allele of rs1051730 was associated with an increased risk of COPD regardless of smoking exposure (pooled OR = 1.26, 95% CI 1.18-1.34, p < 10^-5^). At the genotypic level, the ORs gradually increased per A-allele (OR = 1.27 and 1.50 for GA and AA respectively, p < 10^-5^). Besides, AA genotype exhibited an association with reduced FEV1% predicted (mean difference 3.51%, 95%CI 0.87-6.16%, p = 0.009) and increased risk of emphysema (OR 1.93, 95%CI 1.29-2.90, p = 0.001).

**Conclusions:**

Our findings suggest that rs1051730 in *CHRNA *is a susceptibility variant for COPD, in terms of both airway obstruction and parenchyma destruction.

## Background

Chronic obstructive pulmonary disease (COPD) is one of the leading causes of morbidity and mortality worldwide and results in an economic and social burden which is both substantial and still increasing [1]. It is characterized by chronic inflammation and irreversible airflow obstruction which involves structural changes of the lung [2]. Cigarette smoking is the major risk factor for the development of COPD [1]. The chronic inflammatory process induced by tobacco smoking promotes the structural changes in the small airways and parenchyma [2]. Although COPD is regarded as a consequence of smoking, however, only 10-15% of long-term smokers develop symptomatic airflow obstruction [1,3,4]. This indicates that the genetic factors might contribute to the individual susceptibility. Family studies, case-control studies and the recent hypothesis-free genome-wide association (GWA) studies all support the role of genetic disposition in the development of COPD [5-7].

Several single nucleotide polymorphisms (SNPs) in the gene cluster *CHRNA3-CHRNB4-CHRNA5 *on chromosome 15q25 have been identified to be associated with nicotine dependence [8-14]. These SNPs are rs1051730, rs16969968, rs578776, rs8034191, rs588765, rs12914008, and rs931794. The *CHRNA3-CHRNB4-CHRNA5 *region encodes the subunits of alpha-nicotinic acetylcholine receptor (nAChR), which are expressed not only in neurons but also in other tissues [15]. These receptors play an important role in the lung. They are expressed on structural cells such as bronchial and alveolar epithelial cells [15-17]; and inflammatory cells such as mast cells, neutrophils, monocytes and lymphocytes [18]. Experiments of lung cancer cell line and murine models suggested that pulmonary nAChR might serve as a regulator of proliferation and apoptosis to carcinogens [19,20]. Moreover, the α5 subunit of the nAChR may be involved in modifying the inflammatory response to smoking [21]. The variants in *CHRNA *have been reported to be related to functional consequences, such as altered receptor response to nicotine agonist [15] and altered mRNA levels of *CHRNA5 *in brain and lung tissue [22]. Therefore, these variants might cause biological changes in lung, and contributes to the abnormal responses to smoking stimuli observed in COPD patients. Recent studies, especially results from GWA studies, supported the assumption that this genetic locus is also associated with COPD [6,23-29].

In order to confirm a conclusive relationship between genetic locus and susceptibility to COPD, it is important to determine its impact on the development of COPD and COPD-related phenotypes. Individual studies on the association between nAChR polymorphisms and COPD were of limited sample size and there has been a lack of replication between various studies. From the most recent studies focusing on the association between genetic polymorphisms in 15q25 locus and different subtypes of COPD (airway obstruction and emphysema) [24,25], we summarized the current evidence of variants in *CHRNA*. We also performed a meta-analysis on published case-control studies aiming to confirm the effect of nAChR variants on susceptibility to COPD.

## Methods

### Literature search

Literature search was carried out using electronic databases Web of Knowledge (1990 to June 2011) via library of Fudan University and Medline (1990 to June 2011) by PubMed search engines, with the databases being last assessed on 10 July 2011. The search histories were as follows: (variant OR variation OR genotype OR allele OR polymorphism OR SNP OR single nucleotide polymorphism OR linkage disequilibrium OR haplotype) AND (nicotinic acetylcholine receptor OR nAChR OR CHRNA OR cholinergic receptor nicotinic alpha OR cholinergic nicotinic receptor OR cholinergic receptor OR nicotinic ACh receptor OR nicotinic receptor OR 15q25 OR 15q24/25 OR 15q24-25 OR rs1051730 OR rs16969968 OR rs578776 OR rs8034191 OR rs588765 OR rs12914008 OR rs931794) AND (chronic bronchitis OR emphysema OR chronic obstructive pulmonary disease OR COPD). The reference lists of relevant publications were manually searched for additional studies.

Ethical approval was not required for this meta-analysis.

### Study eligibility and data extraction

Studies were included if they reported the genotype count or allele frequency. The included studies should be a case-control design, with at least two comparison groups, i.e. COPD and controls. Population-based studies were included, but studies base on family or sibling pairs were excluded. Self-reported COPD were not acceptable as a reliable diagnosis, and therefore studies using such diagnostic criteria were excluded. In case of studies containing the same data sets as studies that had been published before, only the study with the largest sample size was included. Data published only in abstract form or from web-site materials were excluded due to the fact that these studies had not undergone peer-review and the inclusion of these studies might introduce bias into the meta-analysis. Case reports, review articles, and textbook chapters were also excluded from the analysis. Two authors independently reviewed the abstracts of all the studies generated by the literature search. The variants, which were reported by more than 5 independent studies, were further meta-analyzed. The full text articles of all eligible abstracts were reviewed and data were extracted by the two authors. Any discrepancies were resolved by discussion with a third author to reach a final consensus.

The extracted data included: (1) publication details including year of publication, title, name of the first author, and country in which the study was conducted; (2) type of study design; (3) sample size; (4) age range, gender and ethnicity of the study population; (5) COPD definition; (6) smoking history of the studied subjects; (7) allele frequency; (8) genotype counts; (9) phenotypes (bronchial obstruction and/or emphysema); (10) methods which were used to investigate polymorphisms. The authors of the included studies were contacted for additional information if there was no sufficient data for analysis.

### Data analysis

A methodological meta-analysis was conducted to determine the overall association between the candidate variant in *CHRNA *and COPD. Data from each case-control study were extracted to construct a two-by-two table in which subjects were classified by diagnostic category (COPD *versus *control) and different genotype or alleles. The odds ratios (ORs) were estimated using the major allele or genotype as the reference group. All the statistical analysis was performed by Review Manager Software 5.1.2 (Cochrane Collaboration, Oxford, UK). The Chi-square test was used to evaluate the presence of statistically significant heterogeneity across the studies and the inconsistency index (*I*^2^) was used to quantify the amount of heterogeneity [30]. If there was no heterogeneity between studies, a fixed-effect model was used. Otherwise, a random-effect model was used. The significance of the pooled OR was determined by the Z test, along with 95% confidence intervals (CI). The presence of publication bias was tested using funnel plots. A p value <0.05 was considered as statistically significance.

## Results

### Description of studies

**Figure 1:**
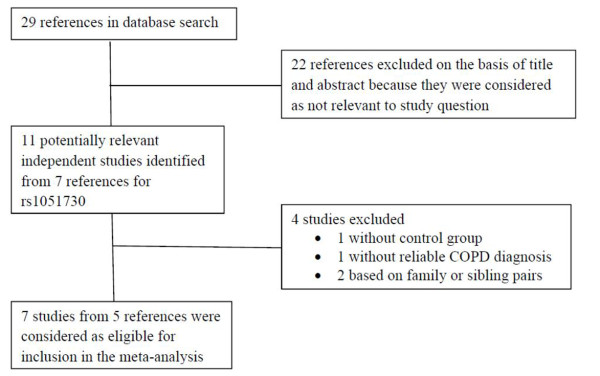
**Study selection process**. COPD, chronic obstructive pulmonary disease.

The initial literature search revealed 29 articles, of which 20 were excluded because their title and abstract were irrelevant to our study question. For all the known variants on 15q25, only rs1051730 was reported by more than 5 independent studies. In the subsequent literature analysis, eleven potentially eligible studies on rs1051730 were identified from the remaining 7 articles [23-29]. The study by Wang et al [29] was excluded because the approach to define COPD was self-reported emphysema rather than spirometer-based criteria. In the Copenhagen City Heart Study, the diagnosis of COPD was based on the ICD coding in the national Danish Cancer, Patient and Death registry [23]. The diagnosis was regarded as reliable because it was registered after hospitalization or outpatient visits. The study by Siedinski et al [28] was excluded because it was a case-only study. The ICGN study and the Boston Early Onset COPD study were family-based analysis, and were also excluded [26]. Finally a total of 7 individual studies from 5 articles met our inclusion criteria. Figure 1 shows the study selection process. In the study by Young et al [27], there were 2 case groups (COPD and lung cancer) and one control group (healthy volunteers). The authors compared COPD group with healthy controls, and with non-COPD subjects including healthy controls and patients in lung cancer group without COPD, respectively. Considering the overlap of the frequencies of acetylcholine nicotinic receptor variants between lung cancer and COPD, the data of comparison between COPD and primary healthy controls was included into the current pooled analysis. Therefore, totally 14897 subjects were included in the current analysis, including 3460 COPD and 11437 controls. Table 1 and 2 summarizes the main characteristics of the 7 included studies. These studies were published between 2008 and 2011.

**Table 1:** Characteristics of studies included in the meta-analysis.

Study and reference	Ethnicity/Country	Age	COPD definition	Smoking history	Methodology of genotyping
Bergen cohort from Pillai et al [26]	NR/Norway	NR	post-bronchodilator FEV1<80% predicted and FEV1/FVC<0.7	a minimum smoking history of 2.5 pack-years	Illumina's HumanHap550 BeadChip
Copenhagen City Heart Study from Kaur-Knudsen et al [25]	NR/Denmark	NR	ICD-8 491-492 ICD-10 J41-J44	NR	TaqMan method
COPDGene study from Kim et al [24]	non-Hispanic white subjects/America	45-80	post-bronchodilator FEV1<80% predicted and FEV1/FVC<0.7	a minimum smoking history of 10 pack-years	TaqMan method
COPACETIC cohort from Lambrechts et al [23]	NR/the Netherlands	50-75	pre-bronchodilator FEV1/FVC<0.7	a minimum smoking history of 20 pack-years	Illumina's HumanHap610-Quad BeadChip
LEUVEN cohort from Lambrechts et al [25]	NR/Belgium	>50	post-bronchodilator FEV1/FVC<0.7	a minimum smoking history of 15 pack-years	iPLEX™ genotyping assay
NETT/NAS cohorts from Pillai et al [26]	non-Hispanic white subjects/America	NR	FEV1 ≤45% predicted and bilateral emphysema on chest CT	cases: former smokers controls: a minimum smoking history of 10 pack-years	Sequenom iPLEX or TaqMan method
New Zealand study from Young et al [27]	Caucasian/New Zealand	Case: >40; Controls:45-80	post-bronchodilator FEV1<80% predicted and FEV1/FVC<0.7	a minimum smoking history of 15 pack-years	iPLEX™ genotyping assay

Abbreviations: NR, not reported; COPD, chronic obstructive pulmonary disease; FEV1, forced expiratory volume in 1 second; FVC, forced vital capacity

**Table 2:** Smoking amounts of COPDs and controls in the included studies.

Study and reference	Pack-years of smoking	
	
	Controls (N)	COPDs (N)
Bergen cohort from Pillai et al [26]	19 ± 13*(810)	32 ± 19*(823)
Copenhagen City Heart Study from Kaur-Knudsen et al [25]	NR	NR
COPDGene study from Kim et al [24]	52 ± 29*(335)	37 ± 19*(507)
COPACETIC cohort from Lambrechts et al [23]	38.0 (28.0-46.2) (295)	38.7 (29.7-49.5)(161)
LEUVEN cohort from Lambrechts et al [25]	42.3 (30.0-50.5) (184)	40.0 (30.0-57.0) (475)
NETT/NAS cohorts from Pillai et al [26]	40 ± 28*(472)	66 ± 30*(389)
New Zealand study from Young et al [27]	40 ± 19*(488)	47 ± 20*(458)

Abbreviations: Data were presented as median (25th - 75th percentiles) for COPACETIC and LEUVEN cohorts, and as mean ± SD for other studies. NR, not reported; COPD, chronic obstructive pulmonary disease, defined by spirometric results; * p < 0.05

### Association between rs1051730 and COPD

**Figure 2:**
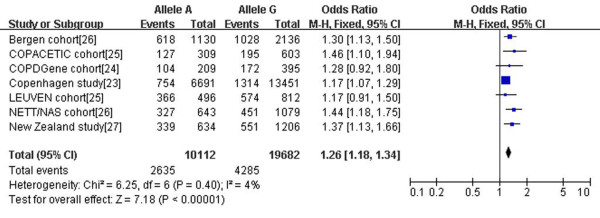
**Forest plot of the pooled ORs for COPD comparing rs1051730 A-allele with G-allele**. The circles in squares and the horizontal lines represent the point estimate and 95% CI, respectively, for each included study; the diamond shape represents the pooled estimate and 95% CI. OR, odds ratio; COPD, chronic obstructive pulmonary disease; CI, confidence interval; df, degree of freedom.

Data from seven independent studies were analyzed for the OR of A-allele using the G-allele as the reference group [23-27]. As the statistical heterogeneity and inconsistency were not found between studies (p = 0.40, χ2 = 6.25, I^2 ^= 4%), a fix-effect model was used for the analysis. The A-allele was significantly more common in COPD subjects compared with control subjects. As shown in Figure 2, the pooled OR of A-allele was increased to 1.26 (95% CI 1.18-1.34, p < 10^-5^). Therefore, the A-allele carriers were more susceptible to having COPD.

According to the comparability of smoking exposure (pack-years) between COPD group and control group, we performed a subgroup analysis. As shown in Table 2, the numbers of pack-years were comparable between COPD group and control group in COPACETIC and LEUVEN cohort. The ORs of both subgroup analyses were similar to the overall estimate (Table 3). Besides, we performed pooled-analysis for 3 included studies in which detailed smoking exposure data were provided, and no associations were observed in terms of smoking amount (pack-years), smoking duration (years) or current smoking status (data not shown).

**Table 3:** Association between rs1051730 A-allele and COPD

Smoking amount	Number of subjects		A-allele vs G-allele effect			**Heterogeneity**		
			
	Control	COPD	OR (95%CI)	Z	p	Chi^2^	p	I^2 ^(%)
comparable	479	636	1.29 (1.07- 1.55)	2.62	0.009	1.34	0.25	25
incomparable	2105	2177	1.35 (1.22- 1.48)	6.12	<10^-5^	0.77	0.86	0

Subgroup analysis was performed according to the comparability of smoking amount between COPD and control group of included studies. Abbreviations: COPD, chronic obstructive pulmonary disease; CI, confidence interval.

Six studies reported the genotype counts. There was no heterogeneity between studies (for all the comparisons, p > 0.05), and analysis was performed under a fix-effect model. At the genotypic level, homozygous AA and heterozygous GA carriers exhibited an increased risk of having COPD compared to homozygous GG carriers (Table 4).

**Table 4:** Association between rs1051730 genotypes and COPD

Genotype	Number of subjects		Overall effect			Heterogeneity		
			
	COPD	Total	OR (95%CI)	Z	p	Chi^2^	p	I^2 ^(%)
GA/GG	1432/1201	6227/6188	1.27 (1.15- 1.40)	4.81	<10^-5^	7.71	0.17	35
AA/GG	438/1201	1621/6188	1.50 (1.30- 1.73)	5.51	<10^-5^	2.02	0.85	0

Abbreviations: COPD, chronic obstructive pulmonary disease; CI, confidence interval.

**Figure 3:**
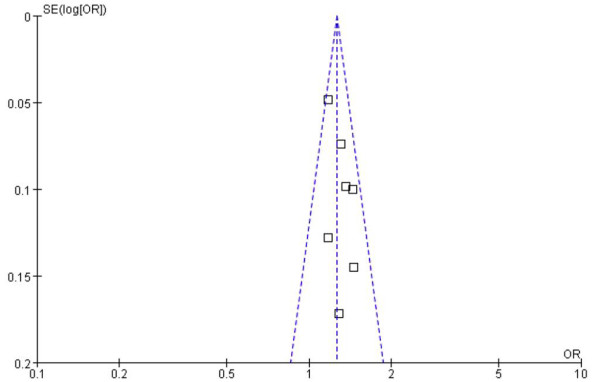
**Funnel plot for the assessment of potential publication bias in rs1051730 variant frequencies**. SE, standard error; OR, odds ratio; COPD, chronic obstructive pulmonary disease.

The funnel plot for the OR analysis of the frequencies of A-allele and G-allele between COPD and control (Figure 3) suggests symmetry, indicating no evidence of publication bias.

### The rs1051730 A-allele carriers showed a reduced lung function

**Figure 4:**
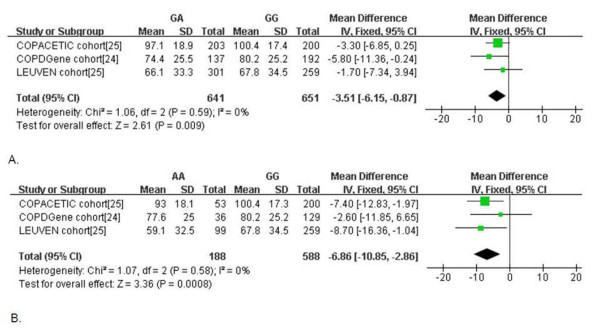
**Forest plot showing the comparisons of FEV_1_%predicted between different rs1051730 genotypes**. A: GA versus GG; B. AA versus GG. The circles in squares and the horizontal lines represent the mean difference and 95% CI, respectively, for each included study; the diamond shape represents the pooled mean difference and 95% CI. FEV_1_, forced expired volume in 1 second; CI, confidence interval; df, degree of freedom.

COPD is a disease characterized with irreversible airflow obstruction which is confirmed by sprirometry. Spirometric results were therefore used to analysis the association between SNP rs1051730 and airway obstruction. Three studies presented forced expiratory volume in 1 second (FEV_1_) values against different genotypes. FEV_1_% predicted values in GA and AA genotype were significantly reduced compared to that in GG genotype, with the mean difference being 3.51% and 6.86% respectively (Figure 4).

### Association between SNP rs1051730 and emphysema

Emphysema is a major pathological change in patients with COPD, indicating tissue destruction. As shown in Table 5, carriers of A-allele had a higher susceptibility for emphysema (OR 1.39, 95%CI 1.16-1.68, p = 0.0005). Similarly, AA and GA genotype both showed an increased risk for emphysema compared to GG genotype. The ORs gradually increased per A-allele (OR 1.37, p = 0.02 and OR 1.93, p = 0.001). Because the definition of emphysema was inconsistent in various studies, the number of subjects included in the pooled-analysis was small, but the associations were still statistically significant.

**Table 5:** Association between rs1051730 genotypes and emphysema

Genotype	Number of subjects		Overall effect			Heterogeneity		
			
	COPD	Total	OR (95%CI)	Z	p	Chi^2^	p	I^2 ^(%)
A-allele/G-allele	379/557	728/1288	1.39 (1.16-1.68)	3.48	0.0005	0.01	0.94	0
GA/GG	219/169	452/418	1.37 (1.04- 1.80)	2.25	0.02	1.91	0.17	48
AA/GG	80/169	138/418	1.93 (1.29- 2.90)	3.18	0.001	0.28	0.60	0

Abbreviations: CI, confidence interval.

## Discussion

Genetic findings suggest that COPD is associated with the chromosome 15q25 region, which includes the *CHRNA5-CHRNA3-CHRNB4 *cluster of cholinergic nicotinic receptor subunit genes [6,12,15,23-29]. Our study only focused on the available case-control COPD studies for this region, and it is the most comprehensive meta-analysis to date on COPD and nAChR genetic susceptibility. We performed both allele-based and genotype-based comparisons. We also had data from the most recent published GWA studies (Bergen and COPACETI cohort [25,26]). Of 7 reported variants in *CHRNA*, we identified rs1051730 with an adequate number of studies for quantitative pooled-analysis. As shown, A-allele of rs1051730 was significantly associated with a risk of having COPD (OR 1.26, 95%CI 1.18-1.34, p < 10^-5^). In addition, AA genotype, compared to GG genotype, showed statistically significant evidence of association with lower FEV_1_% predicted (mean difference 3.51%, 95%CI 0.87-6.16%, p = 0.009) and higher incidence of emphysema (OR 1.93, 95%CI 1.29-2.90, p = 0.001).

There was limited data about the association between the susceptibility to COPD and other variants in *CHRNA*. In the COPD GWA study, rs16969968 (which is in strong linkage disequilibrium with rs1051730) showed a weaker association than rs1051730 [26]. In the same GWA study, rs8034191 was related to a higher risk of having COPD. An association between rs8034191 and FEV1 in emphysema was observed in individual studies [24,26]. In a recently published meta-analysis with the primary objective to identify the variants affecting smoking quantity in chromosome 15q25, 3 data sets with COPD trait were used to analysis the relationship between COPD and rs16969968, rs588765, and rs578776, but only rs16969968 showed a suggestive association [12].

The overlap of nAChR polymorphisms for nicotine dependence and COPD has led to the debate whether polymorphisms in *CHRNA *have a direct effect on COPD or merely mediate smoking habits [8,15,27]. Saccone *et al *[12] reported a weak association between rs16969968 and COPD (OR 1.12, 95%CI 1.01-1.23, p = 0.01) after adjusting for cigarettes-per-day, and the estimate was lower than that for cigarettes-per-day. However, the analysis of Saccone *et al *[12] included self-reported COPD and chronic bronchitis, and this might weaken the association between SNPs and COPD. Results of the current study indicated a stronger association between SNP rs1051730 and spirometer-confirmed COPD, and SNP rs1051730 directly impacts the development of COPD. ORs of SNP rs1051730 for COPD were increased whether the smoking amount was comparable between COPD and control group or not, although the statistical power was stronger when the smoking exposure amount was larger in COPD group than control group (Table 3).

The acetylcholine receptors are expressed throughout the lung and mediate the direct effects of nicotine [31]. Besides regulating cholinergic activities in the airways, recent studies demonstrated that they also played a role in cellular proliferation and inflammatory response in the lung [18,32]. Therefore the nAChRs could not only be correlated to smoking traits such as cigarettes-per-day and smoking addition, but also could be related to other biological effects. Polymorphism of rs1051730 in 15q25 locus represents a synonymous G to A nucleotide exchange in the *CHRNA3 *gene. The genetic variant was correlated to the mRNA expression of nAChRs subunits [11]. At the same time, SNP rs1051730 is in linkage disequilibrium with rs16969968, which changes an amino acid (D398N) in alpha5 nicotinic receptor subunits and alters receptor function [15]. These two genetic variants in *CHRNA3/5 *gene cluster might work together and alter the function of nicotinic receptor in lung tissue and further increase the risk for COPD. Our findings showed that SNP rs1051730 was associated with a reduced FEV_1 _(Figure 4), and this might be explained by the increase in airway contraction and mucus secretion due to the altered receptor function. An association was also observed with the presence of emphysema (Table 5). In another study, rs1051730 was reported to be associated with emphysema severity in ex-smokers [24]. The data was not included into the current pooled-analysis, because the emphysema severity was expressed in quantitive CT values and the genotype counting was not available. It is plausible that the alteration in peripheral nicotinic receptors might have an impact on tobacco induced tissue destruction. To confirm the direct effect of rs1051730 on COPD, further investigation is needed to measure the mRNA and protein expressions of nAChRs subunits in COPD lungs, as well as the impact of SNP rs1051730 on the functional alterations and its role in the development of COPD and in different subtypes of COPD.

COPD is a multi-factorial disease, and therefore, a variety of genes in the biological pathway of COPD might act together in the development and progression of the disease, and single polymorphism might have interactions with other genetic factors. Apart from *SERPINA1 *gene encoding the alpha-1 antitrypsin protein [33,34], a series of genetic factors other than nAChR variants have been identified to influence susceptibility to COPD in 6 previous meta-analysis [5,7,35-38]. These polymorphisms were the Tyr113His and His139Arg in *EPHX1*, *IL1RN *variable number tandem repeat (VNTR) polymorphism, the *GSTM1 *null variant, Ile105Val (rs1695) in *GSTP1*, rs2241712/rs1982073/rs6957/rs1800470 in *TGFB1*, 308GA (rs1800629) in *TNF *and rs1799896 in *SOD3*. Furthermore, another gene *IREB2 *was also identified as a potential gene for COPD susceptibility [39,40]. *IREB2 *and *CHRNA *are both on chromosome 15q25. It was found that IREB2 protein and mRNA were increased in lung-tissue samples from COPD subjects in comparison to controls. However, further investigations are essentially required in order to understand more about the interaction between rs1051730 and other genes (such as *IREB2*) for the pathogenesis of COPD in the future.

Our study has the following limitations. First, our study did not consider gene-by-smoking interactions. In all included studies except the Copenhagen City Heart Study, inclusion criteria on smoking history was similar in case and control group (Table 1); however, the smoking amount were comparable only in COPACETIC cohort and LEUVEN cohort (Table 2). Given the importance of smoking in the development of COPD and the known association between nAChR variants and smoking behavior[11,12,15], future studies with equivalent smoking exposure in case and control subjects are required to address this limitation. Second, we only analyzed part of data of COPACETIC GWA study because the full set of results was not available. Third, the imaging protocols in the detection of emphysema are different in various studies. The number of studies for emphysema is therefore insufficient, because the heterogeneity of the calculating methods for emphysema severity. Fourth, all the subjects in the current meta-analysis are European-descendants. Future studies in Asian and African populations would help to determine the ethnic differences of the relationship between rs1051730 and COPD. Finally, publication bias might have influenced some of our results. Exclusion of case-reports, letters to editors and conference abstracts might have contributed to publication bias. Usually, studies with positive results have a greater chance to be published and studies with inconsistent results of association are usually not published, thus these can potentially increase publication bias in our analysis.

In summary, quantitative meta-analysis identified rs1051730 in *CHRNA *as a susceptibility variant for the development of COPD, in terms of both airway obstruction and parenchyma destruction. A-allele carriers have a higher risk for COPD, and have a lower FEV_1_%predicted and higher incidence of emphysema. However, many questions are still to be answered before establishing nAChRs as an interventional target for COPD. Epidemiologic analysis in different ethnic populations, the mediating effect of smoking exposure, and functional studies should be prioritized for further research.

## Competing interests

The authors declare that they have no competing interests.

## Authors' contributions

JZ participated in the design of the study, carried out the database search, performed article revaluation and data retraction, and drafted the manuscript. HS carried out article revaluation and the data retraction. YGZ performed the statistical analysis. JMQ conceived of the study, participated in the design and coordination of the study and. All authors read and approved the final manuscript.
